# Leveraging Injection Networks to Prevent HIV and Other Blood Borne Infections Among People Who Inject Drugs in Kenya: Design and Rationale

**DOI:** 10.21203/rs.3.rs-7401891/v1

**Published:** 2025-08-20

**Authors:** Matthew J. Akiyama, Hannah N. Manley, Lindsey R. Riback, Chenshu Zhang, Amirhossein Alvandi, Krista Gile, Yun Jiang, Mercy Nyakowa, Nazila Ganatra, Issak Bashir, Ewan Coleman, Jack Stone, Peter Vickerman, Josephine G. Walker

**Affiliations:** Albert Einstein College of Medicine; Albert Einstein College of Medicine; Albert Einstein College of Medicine; Albert Einstein College of Medicine; University of Massachusetts; University of Massachusetts; University of Massachusetts; National AIDS and STI Control Programme (NASCOP); National AIDS and STI Control Programme (NASCOP); Kenya Ministry of Health; University of Bristol; University of Bristol; University of Bristol; University of Bristol

**Keywords:** HIV, hepatitis C, people who inject drugs, LMIC

## Abstract

**Background:**

HIV and Hepatitis C (HCV) are blood borne infections (BBIs) that remain a significant cause of global morbidity and mortality among people who inject drugs (PWID). UNAIDS and WHO have set goals for the elimination of viral hepatitis and HIV as major public health threats by 2030. To achieve these targets, innovative strategies are required among marginalized populations such as PWID, especially in resource-limited countries where coverage of harm reduction services is often limited. The goal of this study is to inform targeted strategies to prevent transmission of BBIs among PWID.

**Methods:**

We will use respondent driven sampling (RDS) to recruit PWID from needle and syringe programs in Kenya. Participants will complete biobehavioral and social network surveys and receive point-of-care HCV, HIV, and hepatitis B (HBV) testing. Participants will return for at least one follow-up visit to complete additional surveys and testing. We will use network data from RDS, egocentric, and viral phylogenetics to identify how highly central PWID contribute to transmission networks and use mathematical modelling to investigate the impact of targeted interventions based on network characteristics.

**Discussion:**

This study will provide important information for policymakers and researchers designing strategies for BBI elimination. Network- and molecular epidemiologic-informed tools to guide targeted strategies may be critical to maximizing the impact of treatment and prevention efforts in resource-limited settings. This approach may provide a durable template for future studies, including prospective assessments of targeted prevention and elimination strategies among PWID, and assist with monitoring elimination progress in resource-limited settings.

## Introduction

There are approximately 14.8 million people who inject drugs (PWID) worldwide, with an estimated 38.8% (or 5.8 million) living with hepatitis C (HCV), 15.2% (or 2.3 million) living with HIV, and 8.4% (or 1.2 million) living with hepatitis B (HBV) [1]. Between 2010 and 2022, annual new HIV infections among PWID decreased by 24% [2]. This is a significant improvement from data from 2016 indicating rising incidence among PWID [3]; however, risk of HIV acquisition among PWID remains 14 times higher than the general population [4]. People living with HCV and HBV are at risk of progression to liver cirrhosis, hepatocellular carcinoma, and liver failure [5]; without effective treatment and prevention interventions, 19 million global viral hepatitis-related deaths are expected by 2030 [6].

HIV prevalence among PWID in Sub-Saharan Africa is lower than the global prevalence (11.2% Sub-Saharan Africa, 15.2% global), and HCV prevalence among PWID in Sub-Saharan Africa is low (20.6% Sub-Saharan Africa, 52.5% global) [1, 5]; however, an increasing number of PWID live with and are becoming HIV- and HCV-infected in this region. Additionally, direct-acting antiviral (DAA) treatment for HCV in many African countries is limited [6, 7] and antiretroviral therapy (ART) uptake for HIV among PWID has been challenging [8, 9]. As such, targeted public health strategies may be an important way to increase the efficiency of preventing transmission of HIV, HCV, and other blood borne infections (BBIs) among PWID in Sub-Saharan Africa. Commonly applied strategies based on evidence from predominantly European and North American settings may not meet the needs of populations in Sub-Saharan Africa due to infrastructural challenges, delays in scale up, or sub-optimal coverage [2, 7, 10]. The implications of delayed treatment and prolonged HIV and HCV viremia in a population with high risk for community transmission is alarming and underscores the need for systematic surveillance and interventions in the context of comprehensive health service delivery systems. However, HIV and HCV surveillance and infrastructure for intervention are underdeveloped in many LMICs, particularly among marginalized populations like PWID.

Conservative estimates place approximately 36,000 PWID in Kenya [1, 11]. Among this population, HCV prevalence has recently been estimated to be 1% in the Western region, 13% in Nairobi, and 22% in the coastal region [12]. The geographic gradation in HCV prevalence is likely attributable to relatively new access to heroin beginning in the 1980s associated with a tourism boom on the Kenyan coast, which gradually spread inland [13]. Kenya introduced needle and syringe programs (NSPs) and opioid agonist therapy (OAT) as part of a nationwide HIV prevention strategy in 2012 and 2014, respectively [13]. NSP sites provide PWID-specific services in line with the WHO recommended programming, including needle and syringe exchange, HIV testing and counseling, HIV treatment and care, prevention and treatment of sexually transmitted infections, and condom distribution, among other services [14]. OAT sites dispense medication for opioid use disorder and provide other services like education and treatment planning, laboratory monitoring, individual and group therapy, and referrals to other services [15]. A recent analysis found that OAT and NSP usage conferred benefits on several different HIV-related outcomes; OAT access was associated with increased ART coverage and viral suppression, and NSP access was associated with lower HIV incidence [16]. The Government of Kenya published its first viral hepatitis guidelines and committed to scaling up DAA therapy in 2017, and have identified PWID as a priority population [17]. Initial data on treatment outcomes among PWID are promising [18, 19]. Modeling data indicate that scale-up of DAA coverage in combination with harm reduction interventions could reduce HCV incidence in Kenya by 90% by 2030 [20], and that DAA treatment for PWID can be cost-effective [21].

Despite the successful implementation of these programs, challenges remain [22]. A recent global systematic review found that Kenya had fairly low NSP and OAT coverage relative to the number of PWID in the country [23] and funding gaps for these harm reduction programs pose obstacles to consistency of care provision and opportunities to expand services [24]. Additionally, challenges with availability and access to commodities for testing and treatment, and financial and policy barriers pose obstacles to greater DAA scale-up and uptake [10]. Recent funding cuts to global health organizations have also drastically impacted clinics and drop-in centers offering HIV and HCV care, substance use disorder treatment, and harm reduction services [25–27].

Social network characteristics play an important role in HIV and HCV transmission among PWID [28–32]. Factors like network size, composition and characteristics, and density have been shown to be associated with multiple behaviors associated with both HIV and HCV risk, including sharing of needles/syringes and other injection paraphernalia [33]. Individuals’ positions within a social network can also be ranked using different measures of centrality such as degree, closeness, betweenness, and Eigenvector centrality. Highly central positions (i.e., being highly-connected at the center of a network) have been linked with HIV and HCV infection, as well as risky injection and sexual behaviors [31, 34, 35]. Little network simulation research has been conducted in Sub-Saharan Africa, and it is unclear how generalizable findings from other settings may be to Sub-Saharan Africa, warranting further investigation in the region.

Geographic factors related to transmission networks also influence HIV and blood borne infection risk. For example, settings of care and harm reduction services, as well as locations of injection hotspots and places to purchase drugs influence BBI risk and transmission [36]. Evidence from studies in other settings indicate that while social network analyses alone may be helpful in identifying individuals for HIV and HCV treatment and reducing community viral load [37], geospatial analyses should also be considered, as BBI transmission may also be clustered at certain locations which could then be targeted for venue-based interventions [29, 30, 32]. Preliminary analyses from our study have also shown geographic variations in social network characteristics [38], further highlighting the need for continued investigation of socio-spatial networks among PWID in Sub-Saharan Africa.

The high transmissibility and genetic diversity of HCV can be leveraged to better understand BBI transmission networks among PWID, as similarity between strains is indicative of transmission linkages. Phylogenetic analyses can be used to model historical relationships between infected individuals and identify individual and geospatial characteristics that account for transmission [39, 40]. Understanding HCV transmission networks allows for identification of PWID who are most likely to receive and transmit HCV and other BBIs. The 2014 HIV outbreak in Scott County, Indiana, in which a cluster of 11 HIV-positive PWID grew to 181 cases within one year [41, 42], demonstrates how understanding of high centrality PWID within injection networks is critical to informing prevention efforts. Over half of the recently HIV-infected PWID in this outbreak belonged to the largest HCV transmission cluster; a better understanding of this transmission network could have been used to better inform prevention strategies that could have prevented this HIV outbreak [41, 42]. Incorporating phylogenetic analysis into traditional social network analyses expands upon existing understanding of transmission relationships and provides additional empirical information on transmission linkages. Social network and phylogenetic analysis can be used in tandem to identify and rank nodes (i.e., individuals) according to various measures of centrality [43]. Identification of central individuals using multiple network measures could be used to more effectively target interventions and to investigate additional factors associated with high centrality.

## Methods

### Study Objectives

The primary aim of the study is to characterize PWID networks, including HCV transmission networks, and identify high centrality PWID. We also aim to determine demographic, behavioral, virologic, and geographic risk factors associated with highly central PWID. We hypothesize having a highly central position will be associated with the greatest risk of HIV and HCV. Furthermore, we will develop mathematical models to examine population-level effects of targeted, network-based interventions to reduce transmission of HIV, HCV, and other BBIs.

### Study Design

We will use respondent driven sampling (RDS) to recruit PWID in Coastal Kenya, Nairobi, and the Western Region. RDS was chosen because it is useful in reaching “hidden” populations and can overcome biases of traditional snowball sampling [44–47]. Additionally, RDS represents one means of understanding connections between participants and is thus useful in finding highly linked people.

### Study Settings

Study settings mirror those of the Testing and Linkage to Care for Injection Drug Users in Kenya (TLC-IDU) study (NCT01557998) [12, 48], which leveraged the emergence of harm reduction programs in Kenya to identify, test, and link PWID to HIV and HCV care, and demonstrate the benefits of these interventions [16]. This study will provide continuity by linking participants from harm reduction to HIV and HCV care, but data collected in this study will also be used to inform new models and strategies for treatment and prevention of BBIs in PWID.

#### Needle and Syringe Programs (NSPs)

We will recruit RDS seeds from and conduct visits at 17 NSP sites in Coastal Kenya (N=8; 3 Mombasa, 3 Kilifi, and 2 Kwale), Nairobi (N=5; 4 Nairobi and 1 Kiambu), and the Western Region (N=4, 1 Kisumu, 2 Migori, and 1 Kisii). NSPs provide injection harm reduction kits (consisting of alcohol swabs, sterile water, sodium hypochlorite solution, and citric acid powder); employ peer educators/navigators who demonstrate safe injection practices, risk-education counseling, and safer sex education; and utilize peer case managers who follow up on individuals who are HIV- and/or HCV-positive. Specified care providers at NGOs in all three study regions have also been engaged to provide PWID-specific services. These sites participate as NSPs and link clients with additional services. The peer navigators maintain a log of all services received by clients in collaborating facilities as per facility protocol, allowing them to track ART, DAA, and other service uptake.

### Study Population and Sampling

We will enroll up to 3500 PWID in Coastal Kenya, Nairobi, and the Western Region. Biometric data capture using iris scanners will be used to prevent double enrollment and to track repeat visits. Participants who test HIV-positive and/or HCV antibody-positive will be eligible to complete up to three follow-up visits in the year following their baseline visit. Additionally, participants who test HIV-negative and HCV antibody-negative will complete a one-time follow-up visit to assess for seroconversion. For each visit, participants will be compensated 400 KSH.

To perform RDS sampling, study and NSP staff will identify individuals who are highly socially networked to serve as seeds and begin peer recruitment chains. Between two and six initial seeds will be recruited from each NSP site. Potential seeds will complete a brief interview with study staff, after which seeds will be selected based on the number of other PWID they know and associate with, previous testing access and results, and length of time spent in each area/neighborhood. Once consented by research staff, these seeds will be instructed on how to recruit up to three eligible peers and offer them a coupon that allows study staff to track recruitment relationships. If the potential recruit approaches study staff with the coupon and expresses interest in participation, they will be screened and consented for the study. Potential recruits need not already be clients at the NSP site at which they redeem the coupon. Participants who recruit their peers will receive 100 KSH for each successful referral.

After enrollment and once all three coupons are distributed, each participant will be provided with up to five tokens and instructed to give these tokens to individuals who they know are already enrolled in the study. Tokens will be used to establish connections between existing study participants, compared to coupons which only allow participants to recruit peers who are not already enrolled in the study. Tokens will allow for better understanding of network connections than the limited tree-structures available in RDS. Individuals who are retained for the follow-up cohort will receive up to eight follow-up tokens to distribute at each follow-up visit. When participants redeem tokens, they will complete a brief survey and receive payment. Participants who provide another participant with a token will receive 50 KSH upon the token’s successful redemption; those redeeming the token will receive 100 KSH. Tokens will allow for visualization of how network members change or stay the same over the study period.

Subjects who are unable to recruit peers will be asked to return their unused coupons (a “buy-back” process, 50 KSH per unredeemed coupon) at the end of each recruitment period. They will participate in a short interview asking about their experience trying to recruit peers and reasons why they were unable to recruit peers. Similarly, participants whose tokens are not redeemed will also complete a buy-back process involving a short survey with questions on how many tokens were given out, whether any tokens were refused, by whom, and what the reasons for refusal were. Participants will receive 20 KSH per token in the buy-back process.

### Eligibility Criteria

Individuals are eligible for inclusion if they 1) are over the age of 18, 2) have ever injected any non-prescribed drugs, 3) have used any non-prescription drugs within the past 12 months, and 4) are able and willing to provide informed consent. Participants will be excluded if they are unable or unwilling to provide informed consent in English or Swahili.

### Procedures

#### Brief Behavioral Survey

A brief behavioral survey will be administered to all participants in English or Swahili by study staff using computers. Domains in the survey include demographics, sexual risk behaviors, alcohol and drug use, IDU-related risk behaviors, and utilization of harm reduction services (Table 1).

#### Social Network Survey

All participants will also complete a social network survey that will provide information on their egocentric network. In this survey, participants will list up to 15 other PWID by name that they know and spend time with (e.g., family and friends, spouses or sexual partners, people that they purchase drugs from, people they inject drugs with, and ‘injection doctors’ (individuals located in injection venues who inject for others [55])) and provide information on their relationships and interactions with these individuals. This information relates to injection practices (date of last injection with this person, equipment and drug sharing); sexual behaviors (frequency of sexual contact, condom usage); and HIV, HBV, and HCV (testing and treatment status).

#### HCV Testing

After obtaining study consent and completing the behavioral survey, all participants without a recent (i.e., within the prior three months) HCV test result reported in the DIC registry will receive rapid HCV antibody testing (Abbott, Bioline HCV). Confirmatory viral load testing (Cepheid, GeneXpert) will be done at the National Public Health Laboratory in Nairobi for those who test HCV antibody positive. Participants undergoing confirmatory testing then return to the study site to receive their results, counseling, and referral to treatment. All testing procedures follow Government of Kenya standard of care [56]. For individuals with detectable HCV RNA, the sample will be used to perform next-generation sequencing (NGS) and phylogenetic analysis.

##### HCV Sequencing and Analysis.

Total nucleic acid (TNA) will be extracted from all specimens using 200 μl of plasma on KingFisher Pure Viral TNA Kit (Roche, Indianapolis, IN). Each RNA sample will be eluted in 50 μL of Elution Buffer and 21 μL will be subjected to DNA synthesis performed with one-step RT-PCR kit (Invitrogen, Thermo Fisher, Waltham, MA), targeting the hypervariable region (HVR1) of the HCV genome. Secondary Barcode nested PCR reactions containing unique 10-mer barcodes in both the reverse and forward HVR1 primers will be performed using PerfeCTa Reaction Mix (Quanta Biosciences, Beverly, MA). The amplicons will then be indexed with 8-mer indexes and adapters (IDT- Integrated DNA Technologies, Inc., Coralville, IA) appropriate for sequencing, clustering and demultiplexing on the Illumina MiSeq instrument (Illumina Biotechnology Company, San Diego, CA). The products will be purified using Ampure XP (Agilent Technologies), purity will be checked and quantified on Tape Station Instrument (Agilent Technologies, Santa Clara, CA). The quantified amplicons will be normalized and mixed to generate a 24 plex library including 2 negative controls. The library will be diluted to 10 pmol and sequenced using paired-end read protocol on Illumina MiSeq and v2 Nano chemistry. The library will be automatically de-multiplexed on the MiSeq instrument. Paired demultiplexed Illumina MiSeq output in form of FASTQ files will be uploaded onto the cloud-based portal called GHOST Global Health Outbreak and Surveillance Technology (GHOST) – Version 0.9.1 (cdc.gov), which provides a set of bioinformatics tools that apply quality control measures and analyze NGS data.

#### HIV Testing

All participants without a recent (i.e., within the prior three months) HIV test result reported in the DIC registry will also undergo rapid HIV testing conducted by study staff trained and certified in HIV counselling following NASCOP/Government of Kenya standards [57, 58]. Due to changes in Kenya’s testing guidelines [59], testing used Abbott Determine HIV-1/2 and Abbott First Response HIV-1/2 RDT kit for those with a reactive Determine test May 2022 to February 2025, and Trinscreen from March 2025 onward. Once results are ready, staff will inform participants of their results and conduct post-test counselling. The participants will be referred to the NSP site for appropriate HIV care referral.

#### HBV Testing

Anti-HBV rapid testing (Abbott, SD Bioline HBsAg) will also be provided to all enrolled participants without a recent (i.e., within the prior three months) HBV test result reported in the DIC registry. All tests will be conducted per recommended Kenyan National Guidelines for the Control and Management of Viral Hepatitis [56].

#### Positive Cohort Follow-Up

Participants who test HIV- or HCV antibody-positive at baseline or had a recent positive test recorded will undergo three follow-up visits at four-month intervals in the year following their baseline visit. Participants are given a one-month grace period before and after the four-month mark in which they can complete the visit. Participants who miss a visit are still eligible to complete later follow-up visits. Peer case managers at each site will assist study staff with recalling participants for their follow-up visits by conducting outreach at commonly frequented injection venues. At each of the three follow-up visits participants will complete biobehavioral and social network surveys with updated time frames, and will receive HIV, HCV, and HBV testing.

#### Negative Cohort Follow-Up

Due to resource limitations, it will not be possible to conduct multiple follow-ups for all participants. Following completion of the positive cohort follow-up visits, we will conduct one additional follow-up visit for individuals who test HIV- and HCV antibody-negative during their baseline visit to determine seroconversion with HIV, HCV, and/or HBV. Biobehavioral and social network data will also be collected during these follow-ups.

#### Qualitative Survey

A qualitative survey will be conducted to elicit knowledge, attitudes, and behaviors regarding HCV and associated risk factors among participants found to be highly networked and among participants who report frequenting “base camps” or “dens.” Approximately N=40 participants will be interviewed. Trained staff will audio-record the interviews, which will then be transcribed. Transcripts will then be imported into Dedoose software (Hermosa Beach, CA) for coding and analysis. These interviews will expand upon quantitative data related to social network composition and reported risk behaviors. Additionally, they will provide insight into the acceptability and feasibility of targeting these individuals for HCV treatment and leveraging them to provide PWID-focused HCV and HIV harm reduction education and services.

The survey instrument will be semi-structured and designed using the Rhodes Risk Environment Framework [60, 61] as a theoretical framework. Key informant interviews with PWID who are determined to be highly central in injection networks will be used to enhance information about HCV as well as HIV transmission and prevention. The social and cultural environment could affect the use of prevention practices, which are often mediated by key factors such as stigma, violence, and discrimination. Peers and norms are also external factors that could influence knowledge, attitudes, and behaviors. Lastly, economic factors could prevent changes in practice of harm reduction, as many PWID may not have the resources for transportation to seek clean needles at NSPs and may turn to injection doctors or other sources. These characteristics will be determined in qualitative interviews and applied to theories of behavioral change to ascertain how to create interventions that will successfully lower the risk of HIV and HCV infection/re-infection.

### Ethical Considerations

This study was approved by the Albert Einstein College of Medicine Institutional Review Board (IRB# 2021–12928) and the Kenyatta National Hospital Ethics and Research Committee (ERC; University of Nairobi; IRB# P856/10/2021). Formal informed consent will be obtained from all study participants and participation is strictly voluntary.

### Network Analyses

We will use three layers of network data to characterize the networks of PWID enrolled in our study: 1) Recruitment network, 2) Phylogenetic network, 3) Egocentric network.

#### Recruitment network

RDS network data are restricted by the RDS tree-based structure, which will look similar regardless of the degree of clustering in the network. RDS data alone therefore provide little network information as only ties used for recruitment are observed in the data. While it is possible to make inference about clustering based on homophily on observable characteristics (e.g. are infected population members more likely tied to other infected population members?) [62], it is not possible to infer endogenous clustering – the inherent tendency in social networks for friends-of-friends to be friends. Nonetheless, such clustering is an important feature of disease transmission in networks.

We will use token data to make inferences about the presence and extent of endogenous clustering in these social networks. We will also use this data to infer network structures and compare them to information available in this dataset, but difficult to collect elsewhere, including the distance of GHOST sequence information, and direct reports of social networks. We anticipate this token approach will provide a practical low-cost method to improve the availability of disease-relevant information in RDS data. Token data will also give more complete data from which to estimate network centrality. We will measure degree, closeness, and eigenvector centrality on these RDS coupon and token observed network data to compare to other critical nodal features as well as to centrality based on the other network data sources.

#### Phylogenetic network

NGS will be applied to the HVR1 of the HCV genome to perform phylogenetic analysis for participants with chronic HCV. HVR1 is the most variable and thus the most commonly used in molecular epidemiologic studies to detect clusters of persons infected via transmission events [63]. To visualize linkages in detail, all haplotypes with frequency >2 will be used to generate alignments using CLC Genomics Workbench v11 (QIAGEN Aarhus A/S). The patristic distances (i.e., genetic distance between two sequences in a phylogenetic tree measured in nucleotide substitutions per site) included the pairwise intra-person (two sequences from one individual) and the within-genotype inter-person (two sequences from two different individuals) distances. Cutoffs to phylogenies will be assigned using the intra-person distances, and sequences from different individuals with patristic distances falling below these cutoffs will be interpreted as transmission pairs or clusters. A transmission network that represents the genetic relatedness among HCV in participants will be built using the CDC developed web-based (https://webappx.cdc.gov/GHOST) GHOST, hosting a sequence cleaning algorithm for HVR1 NGS data, and the threshold method for detection of HCV transmission [63].

#### Egocentric network

For further network analysis, egocentric social network data collected through the social network surveys will be analyzed using a probabilistic linkage model. This analysis will allow for examination of differences between RDS/token networks and self-reported social networks, potentially identifying individuals that were missed by the RDS process.

### Additional Planned Analyses

Baseline and follow-up demographic and clinical information will be used to assess demographic, behavioral, structural, virologic, and geographic risk factors associated with positions of high centrality; additionally, we will assess associations between centrality measures and HIV, HCV, and HBV disease status. To assess centrality, we will create a unified network using egocentric social network data, RDS data, and token data; and use results from the GHOST analysis to examine how transmission occurred within these other networks. Tabular analysis will be used to explore bivariate associations between these characteristics, risk behaviors, and the presence of HIV/HCV co-infection or multiple-strain HCV infection. Factors strongly associated with having high centrality with *p*-values less than 0.2 will be considered for inclusion in multivariable models. Because RDS sample is not a random sample, we will conduct weighted regression analyses by using RDS weights, which are calculated as inverse of self-reported network size [64].

To evaluate the effect of having high centrality on transmission and acquisition of blood borne infections, cox proportional hazard models (adjusting for host demographic, risk behavior and clinical factors) will be used to estimate the hazard ratios for associations between HIV/HCV co-infection, multiple-strain infection, and other factors associated with having high centrality on the probability of incident blood borne infections. Incidence rates per 100 person-years of HIV and HCV were estimated to be 2.5 and 6.3 in the coastal region, 1.6 and 2.3 in Nairobi, and less than 1 in the Western region, based on previous data [12].

Individual transmission events defined through GHOST will also be studied to investigate factors associated with directionality of HCV transmission. A global meta-analysis of the prevalence of HIV, HCV, and HBV among PWID showed that HIV was more prevalent among women who inject drugs in some regions, including Sub-Saharan Africa [65, 66]. Participants from another qualitative study reported that individuals in drug dens referred to as “injection doctors” may play a role in unsafe injections and could be highly central vectors of transmission [55]. Specific details of these relationships warrant further investigation and will be explored through qualitative interviews. Additionally, if there are other phenomena that fall outside of the models, in-depth qualitative analysis will be conducted to further elucidate these factors.

Intra-host viral heterogeneity via NGS will be utilized to estimate the duration of time since transmission, enabling the outlining of temporal trends of where initial HCV infections were established and the way in which PWID have migrated over time [67]. Understanding migration patterns leading to movement inland may help to prevent further transmission in the Western region, which has thus far been relatively unaffected by HCV. This understanding of PWID migration may also be useful in informing HIV treatment and prevention efforts.

Lastly, an agent-based model of the transmission of HIV and HCV among PWID will be developed in the Python programming language. The model will be calibrated to the behavioral and social network surveys, disease statuses, and previously published population level data. By reproducing the structural and dynamical properties of the network of injecting partnerships, the model will replicate the disproportionate role that some individuals play in the transmission of disease. The model will be used to predict the impact of different strategies to target interventions (e.g. HCV/HIV test and treat, OAT and NSP).

## Discussion

Understanding injection networks among PWID is a crucial component in preventing HIV, HCV and other blood borne infections. This study will provide a novel characterization at the national level of an HCV epidemic among PWID and elucidate the dynamics of injection drugs use as an underappreciated risk factor for HIV and other blood borne infection transmission in Sub-Saharan Africa. The estimated overall HCV prevalence among PWID in Kenya is low compared with the global average [5, 68], which makes Kenya a key country to model the impact of a targeted strategy on HIV prevention and HCV elimination.

Our study leverages social network and phylogenetic analyses to improve our understanding of HIV and HCV transmission risk among PWID. Given the existing knowledge of social network factors and their relation to HIV and HCV transmission risk [33, 43, 69–71], the addition of phylogenetic analysis in our study will further bolster this understanding. Network analyis using GHOST could be implemented in public health labs to analyze these data making these results actionable in reasonable time periods so that outbreaks could be stopped in early stages, reducing the extent of their spread. Exisiting modeling studies [41, 42] have shown that identifying and intervening on highly central PWID has the potential to reduce transmission and improve the effectiveness public health interventions; however, these studies have been limited to higher-income countries, and little research in this area has been conducted in Sub-Saharan Africa.

This study capitalizes on and benefits from existing infrastructure and collaborations [12, 48]. We have successfully piloted the proposed phylogenetic analysis [12, 72], demonstrating both the feasibility of conducting such an analysis in this population and the potential utility of using phylogenetics to identify and study transmission clusters. Additionally, this study benefits from the Government of Kenya’s commitment to HIV and HCV reduction efforts among PWID and will take advantage of the high utilization of Government of Kenya programs by PWID to help in reaching this “hidden” population.

The qualitative element of this study will address significant gaps in the understanding of BBI prevention and treatment at the physical, social, economic, and policy levels [60, 61] in this population. There is little data on the acceptability and feasibility of providing HCV and HIV harm reduction education and services to high risk PWID, particularly among PWID in Sub-Saharan Africa. The potential significance of this research is in its ability to inform what is known about setting-specific HCV and HIV risk factors and validating the importance of HCV phylogenetic analysis to inform treatment and prevention strategies in the region.

## Limitations

The assertion that individuals who are highly central will be at high risk of receiving and transmitting HIV, HCV and other BBIs may not be evident in this population of PWID. Moreover, the association of HIV/HCV co-infection, multiple-strain HCV infection, or other factors may not be able to be demonstrated in this sample due to sample size constraints. Additionally, though we believe RDS to be the best sampling method for this work, it may also introduce some bias to the study. For example, though RDS assumes recruiters will pass their coupons and tokens to network contacts, it is possible that they may pass the coupon off to a stranger (e.g., someone visiting the NSP site at the same time). Coupon distribution behaviors (e.g., who is approached, why and how they are approached, who accepts and why they accept) may also vary between individuals, leading to undocumented variation. In highly clustered populations, it is also possible that the RDS sample will not mix well and draw from the full target population with the expected proportions. This could lead to undue influence of the seeds on the final sample. This method of recruitment by referral also naturally biases selection towards individuals with a larger number of contacts. Individuals with no contacts will not be represented in the sample. Additionally, given that each referral chain starts at a single location, the geographical distribution cannot be expected to be representative of the whole population of PWID. PWID living close to a seed location are more likely to be included. Additionally, those living far away from an NSP site may be less likely to return a coupon.

## Conclusion

In summary, we aim to meet a critical need by identifying new molecular epidemiologic tools to inform development of national strategies that maximize the efficiency of prevention efforts for HIV, HCV and other BBIs. We expect this study to provide essential information for researchers and policy makers seeking to identify key priorities and strategies for BBI prevention in settings where HIV and viral hepatitis epidemics are converging among PWID. In addition to providing evidence with immediate relevance to policy, modeling the impact of interventions focused on highly central PWID will provide a roadmap for prospective targeted HIV prevention and HCV elimination strategies with the ultimate goal of ending the HIV epidemic and HCV elimination among PWID in LMICs with the potential for broader global relevance.

## Figures and Tables

**Table 1:** Behavioral Survey Domains

Domains	Visit(s)
Sociodemographics	0
Housing	0, 1, 2, 3
Location and travel	0, 1, 2, 3
Substance Use Behaviors [49, 50]	0, 1, 2, 3
Sexual Risk Behaviors [49]	0, 1, 2, 3
History of HIV, HCV, and HBV testing, diagnosis, and treatment	0, 1, 2, 3
NSP utilization	0, 1, 2, 3
History of OAT	0, 1, 2, 3
Healthcare Needs and Access [51, 52]	0, 1, 2, 3
Quality of Life (EQ-5D-5L) [53]	0, 1, 2, 3
Substance Use Stigma Mechanism Scale, Enacted Stigma subscale [54]	0, 1, 2, 3
Violence	0, 1, 2, 3
Incarceration history	0, 1, 2, 3
